# Knowledge of pastoralists on livestock diseases and exposure assessment to brucellosis within rural and peri-urban areas in Kajiado, Kenya

**DOI:** 10.12688/f1000research.20573.1

**Published:** 2019-11-13

**Authors:** Joshua Onono, Penina Mutua, Philip Kitala, Peter Gathura

**Affiliations:** 1 Department of Public Health Pharmacology and Toxicology, University of Nairobi, Nairobi, 00625, Kenya; 2 Ministry of Agriculture Livestock Fisheries and Irrigation, Meat Training Institute, Athi River, Private bag, Kangemi, Nairobi, 00625, Kenya

**Keywords:** Qualitative risk assessment, Zoonoses, Pastoralism, Livestock, Kenya

## Abstract

**Background:** Livestock diseases impact the livelihoods of pastoralists. Brucellosis, a neglected zoonotic disease is highly prevalent in this system with an estimated 16% of livestock population in sub-Saharan Africa infected with the disease. The objective of this study was to assess knowledge of livestock diseases and the risk of exposure to brucellosis among pastoralists living in Kajiado County of Kenya.

**Methods:** The study sites included pastoralist communities living in rural and peri-urban areas within the County. Both primary and secondary data were collected using participatory methods including pairwise ranking, proportional piling and probing and a review of the published literature. Exposure risk assessment was conducted according to the CODEX Alimentarius framework: Hazard identification, hazard characterization, exposure assessment and risk estimation.

**Results:** According to pastoralists, livestock diseases that frequently occurred in their flocks and herds were contagious caprine pleuropneumonia, lumpy skin disease and foot and mouth disease; but zoonoses, including anthrax and brucellosis, were also mentioned during focus group discussions. Potential pathways of exposure to brucellosis and other zoonoses included consumption of unpasteurized milk, handling infected aborted materials without protective measures and consumption of raw meat and raw blood. Consumption of unpasteurized milk and handling infected aborted materials without protectives were linked with high risk of exposure to household members living in rural areas, with the risk level within the peri-urban areas ranked very low to low for most of these risk practices.

**Conclusions:** The results call for enhanced public education targeting vulnerable groups to mitigate risks of disease spread and other impacts of brucellosis within the affected pastoralist production systems.

## Introduction

Brucellosis is a neglected bacterial zoonosis with global distribution, and it has been reported in 56 countries with more than 500,000 new human cases reported annually ( Christou, 2011; Seimenis *et al.,* 2006). The disease is prevalent in pastoralist systems within developing countries and it has been associated with increased public health concerns, especially in the Mediterranean region, western Asia, Latin America and other parts of Africa ( Corbel, 2006; Gwida *et al.,* 2010). In sub-Saharan Africa, about 16% of all livestock raised within the region are estimated to be infected with brucellosis ( Kiambi, 2012). Similarly, several studies have identified *Brucella* species including *Brucella abortus, Brucella melitensis* and *Brucella suis* in different parts of Africa, with isolates which are closely related to strains circulating in the Mediterranean basin ( Ducrotoy *et al.,* 2017; Lounes *et al.,* 2014).

In Kenya, brucellosis was first reported in 1916 with the first case confirmed in a laboratory in 1931 ( Njeru *et al.,* 2016). To date, animal and human brucellosis is reported annually especially from pastoralist production systems ( Kiambi, 2012; Maichomo *et al.,* 2000; Richards *et al.,* 2010). Furthermore, Brucella species have been isolated from both wild and domestic animals ( Muendo *et al.,* 2012; Oomen, 1976). *B. melitensis* and *B. abortus* have also been isolated in human patients from different parts of the country ( Njeru *et al.,* 2016). According to results of a systematic review of brucellosis in Kenya, the estimated national sero-prevalence was about 3.0%, ( Njeru *et al.,* 2016). This high sero-prevalence in humans is reported to occur in several counties including Kajiado, Marsabit, Turkana, Machakos and Garissa, which are counties mainly inhabited by pastoralists ( Ogola *et al.,* 2014). However, a low sero-prevalence has been reported in Kiambu, Naivasha, Busia and Nairobi counties, which are counties where the predominant production system is intensive and semi-intensive livestock production systems ( Njeru *et al.,* 2016; Osoro *et al.,* 2015).

The pastoralist communities are highly dependent on livestock for supply of their household nutritional needs, besides their economic and social utilization ( Bechir *et al.,* 2012; Zinsstag *et al.,* 2006). However, some cultural practices inherent in these communities such as consumption of unpasteurized milk, raw meat and blood, contribute to increased risk of transmission of brucellosis from livestock to humans ( Adesokan *et al.,* 2013; John *et al.,* 2010). Indeed, other practises such as increased mobility of pastoralist communities with their livestock in search for pasture and communal watering grounds also contributes to the spread of infectious diseases in animal populations.

Various studies have shown varying degrees of knowledge about zoonoses, especially brucellosis, amongst populations. This level of awareness is thought to significantly contribute to the control of spread of such infections ( Lindahl *et al.,* 2015). Likewise, lack of awareness on such infections may delay patients from seeking health care in time ( Kansiime *et al.,* 2014), thus enhancing spread of infection amongst populations. Some studies have shown higher level of awareness about brucellosis among the educated when compared to the non-educated populations ( Lindahl *et al.,* 2015). In their study, increased awareness on brucellosis was also reported among the agro-pastoralist population when compared to the group of nomadic pastoralists and farmers who engaged with veterinarians on service delivery for their animal health problems ( Kansiime *et al.,* 2014; Lindahl *et al.,* 2015). A number of studies have also shown that while the majority of the population have heard about brucellosis, only a small percentage of the farming population have knowledge of how the disease is transmitted and its manifestation both in man and animals ( Adesokan *et al.,* 2013; Ljung, 2013).

In Kenya, previous studies conducted have focused on determining prevalence and risk factors associated with spread of brucellosis both in livestock and human populations ( Kang’ethe *et al.,* 2007; Osoro *et al.,* 2015). However, there is paucity of studies that reports associations of these risk practices by farming communities to exposure of household members to infections with brucella from the livestock they keep. This study examined the role of pastoralist practices and behaviour in disease management that increases their risk of exposure to brucellosis from their livestock. The findings of this report are useful in understanding why brucellosis continues to persist within pastoralist communities despite the increasing knowledge amongst both livestock and human health professionals on the available control strategies, including vaccinations and improved breeding management through artificial insemination, which have been used to control the disease in other parts of the World.

## Methods

### Study design and area

This was a descriptive study that examined knowledge, attitudes and practices of the pastoralist community living within selected rural areas in Kajiado and another group who were living within the peri-urban areas. The study was carried out in August 2015. These communities were selected to compare the risk practices of exposure to zoonoses between those who still practiced pastoralist livelihoods system within the county, and another who had transitioned to peri-urban livelihoods systems, but were still bound by cultural ties based on the community customs and traditions. Kajiado County is predominantly inhabited by the Maasai community, which still have members who practices nomadic pastoralism. However, there are immigrants from other communities in the county, with different cultural orientations from those of the Maasai. These have mostly inhabited the peri-urban areas within the county.

**Figure 1.**
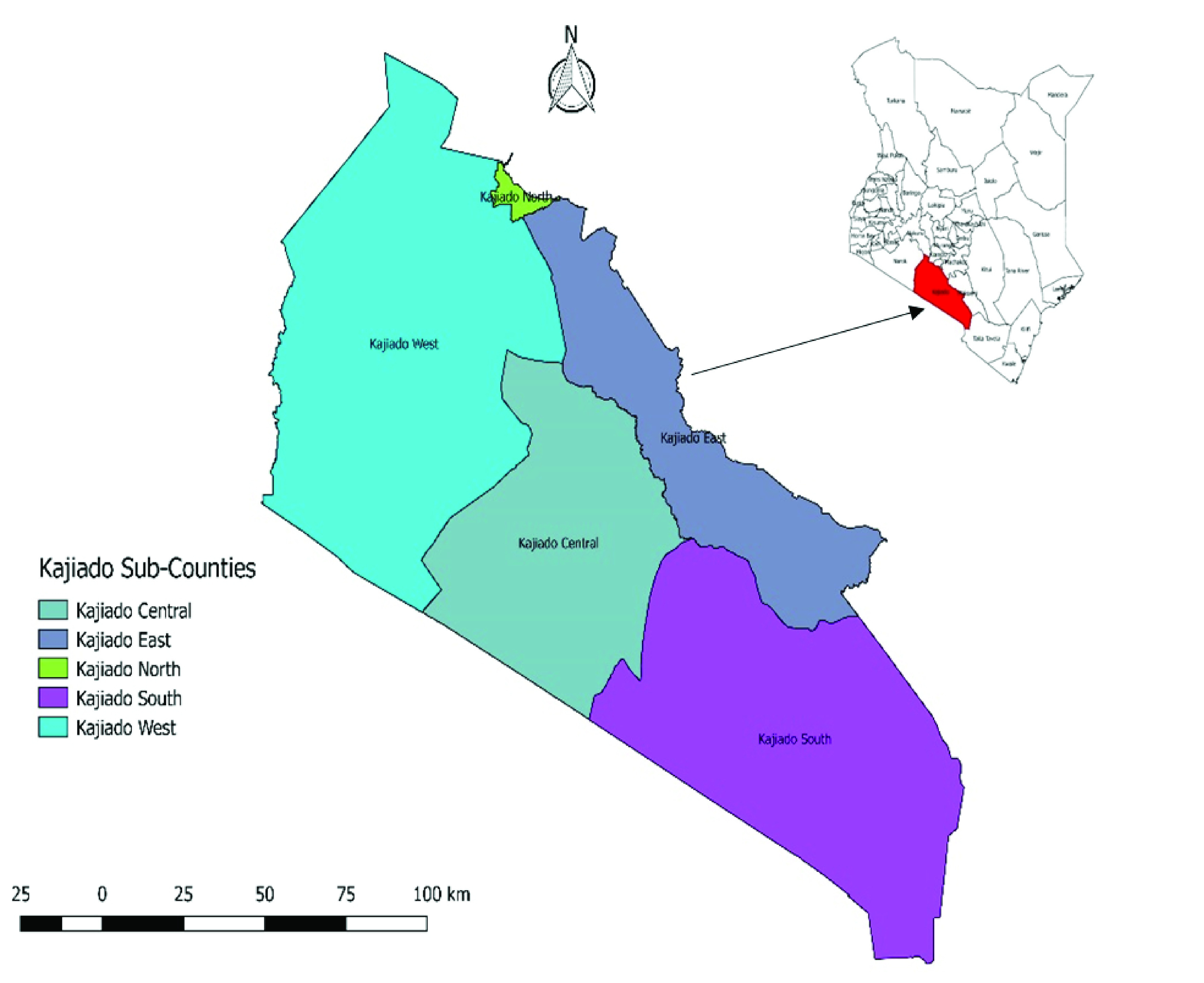
Map of Kenya showing administrative regions in Kajiado County.

Kajiado County has two main livestock production systems: nomadic pastoralism and mixed livestock and crop farming systems (agro-pastoralism) practiced around up-coming urban centers. The county was therefore stratified in two strata, based on these livelihood systems. The study sites were selected through a two-stage sampling process. The sub-counties where the study was carried out were first selected purposively due to available logistical arrangements for accessing the pastoralists, and administrative wards where interviews were carried out were selected from these sub-counties through a random selection process using a lottery method where all wards in each sub county were listed in different pieces of paper, which were then rolled up and placed in a box. From each box two pieces of paper were drawn sequentially, and names of the selected wards recorded. For each of the selected sub-counties, all administrative wards had an equal opportunity of being selected to be included in the study through a process of sampling without replacement. The process was guided by the research team with assistance from a local government extension agent in Kajiado who was familiar with geography of the area and Maasai culture and language. Two sub-counties were selected from this process: Kajiado Central representing the nomadic pastoralism system and Kajiado East representing the peri-urban communities ( Figure 1). In both sub-counties, the number of administrative wards were five. The wards included Purko, Ildamat, Matapato South, Matapato North and Dalalekutuk in Kajiado Central sub county, while for Kajiado East, these were Poka, Imaroro, Kaputei North, Kitengela and Oloosirkon/sholinke wards. Two wards were selected from each of the two sub counties. For Kajiado Central, the selected wards were Matapato North and Matapato South, while in Kajiado East; Kitengela and Kaputei North wards were selected.

### Selection of study subjects

Administration officers (village elders) from each of the selected wards were recruited to help with mobilization of study participants to attend focus group discussions. The participants who were invited to the group discussions were drawn from different parts of the wards where the village elders represented. Each group comprised of between 8 to 11 participants who were identified by village elders to have lived in the area for several years, and who were either of male or female gender were recruited for the study, while anyone who was non-Maasai speaking were not invited for the discussion. This was considered to comply with group composition description of 6–12 participants for a group discussion as was described by Dilshad & Latif (2013), where individuals with a certain characteristics are brought together by a researcher to explore attitudes, perceptions, feelings and ideas about a topic. The local veterinary personnel were also recruited to the study to serve as key informants as well as in assisting during the focus group discussion sessions to triangulate the information on livestock diseases that participants identified. A total of six groups comprising both male and female participants and two groups comprising each gender separately were conducted to explore any differences in household and farm practices with regard to handling livestock and their products which could be gender specific, especially for a patriarchal society like the Maasai.

### Data collection

Two focus group discussions were conducted in each selected ward led by a member of the research team with support from local government extension personnel who was conversant with the Maasai culture and language. The group discussions were held in homesteads of local village elders where community leaders would always meet to resolve communal problems or under makeshift structures which were also used as places of worship by the community. Secondary data on the other hand, was obtained through a review of surveillance data from previous published studies and grey literature sources. Published participatory methods including proportional piling, pair wise ranking and disease matrix scoring were used for data collection during the focus group discussions ( Catley, 2006; Catley *et al.,* 2012). Briefly, pairwise ranking is a structured method of prioritizing a list of factors for decision making through consensus, where each box on a matrix represent an intersection of two constraints. For each pair of listed diseases, participants determined which of the two was most important through consensus and the important disease was marked in the appropriate box. The procedure was repeated until all boxes in the matrix were filled. Diseases were then ranked according to number of times they appeared in the matrix. Proportional piling involve allocating percentage scores to identified list of factors that have been identified by a group of people through consensus. Disease impact matrix scoring employed proportional piling method to estimate effects of livestock diseases on production parameters as perceived by pastoralists. For this case a total of 100 beads representing different weights for impact of livestock diseases were applied. Production parameters assessed included weight loss, mortality, loss in milk yield, and abortion rate. This was based on how participants perceived the disease to impact on these production parameters. The production parameters were entered on the left column of the matrix, while diseases were entered on top of the matrix. The next step involved allocating scores to diseases according to how pastoralist perceived them to impact on each production parameter. This was done sequentially focusing on one production parameter each time by allocating scores across the disease columns until all the rows of the matrix were filled. The scores allocated for each production parameter were then summed to give an overall score. The results obtained were probed to obtain reasons for the observed pattern.

The guiding questions during these sessions included: list of livestock diseases in order of their perceived importance, and what is the effect of listed diseases on livestock production parameters including milk yield, mortality rates, morbidity rates, and abortion rates. For the risk assessment, both primary and secondary data were collected through use of the questionnaire guide during the focus group discussions, and through a review of published and grey literature sources (see extended data ( Onono *et al*., 2019)). Primary data were collected on participants’ knowledge on brucellosis in animals and humans, modes of transmission of brucellosis from animals to man and the potential exposure factors contributing to spread of brucellosis in man. The data were recorded in flip charts, while the research assistant would take additional notes that participants provided to support their argument on the types of choices they made during discussions. The participants were asked whether they knew about brucellosis in animals and in man, what were the symptoms of brucellosis in animals, the ways through which people could acquire brucellosis, factors that contributed to spread of brucellosis in man, disease management in animals and what were their perceptions on the impact of human brucellosis. The factors identified were useful in exposure assessment and estimation of risk of brucellosis infection among the population under study. The literature review was based on studies that had reported presence of the disease (hazard identification) through detection with either immunological or molecular methods within pastoral herds and flocks in Kajiado and neighboring pastoral areas. The search was done in electronic databases including PubMed, Medline and CABI direct, and the search terms included “brucellosis, *Brucella & melitensis, Brucella & abortus*, livestock, Kenya”. Data were also obtained from grey literature sources including published thesis and reports. The inclusion criteria for studies were those which were conducted across all farming systems in Kenya, while exclusion criteria was any other study on brucellosis which was not done in Kenya. Hazard characterization was based on the livestock species from which the hazard was detected in biological samples through either immunological or molecular methods. Before the focus group discussions were conducted, participants were asked if they accepted to participate in the study through a written consent statement which was read to them, and those who accepted to participate were asked to provide their written consent by signing an attendance form. Further ethical clearance for the study was obtained from the University of Nairobi, Faculty of Veterinary Medicine ethics and biosafety committee (FVM: BAUEC/2015/138). 

### Data management and analysis

Data on scores and ranks obtained during focus group discussions were entered in Microsoft Excel software version 2010. Data for various questions which were obtained from the focus group discussions were analyzed using descriptive statistical methods and results presented as frequencies, while scores were analyzed through non-parametric methods using GenStat Statistical package 13th edition ( VSN International, 2011). For the scores, the analysis aimed at measuring the level of agreement amongst groups of the FGDs using Kendall’s coefficient of concordance, while Kruskal Wallis One Way Anova was also used to test whether the average scores obtained for each disease as ranked by participants were significantly different from zero. The impact of the listed diseases on the various production parameters: herd/flock milk yield, abortion rates, growth rates (weight gain), and mortality rates were presented as averages.

Qualitative risk assessment was done according to the CODEX Alimentarius guidelines ( FAO & WHO, 2009). The exposure assessment to infection with brucellosis through the various pathways was determined based on the scores allocated for the likelihood of occurrence of these events in the community by the participants. A high risk score of 5 was allocated when the event occurred very often in the surveyed community; medium risk score of 4 was allocated if the event occurred regularly; low risk score of 3 was allocated if the event ever occurred but was very rare; very low risk score of 2 was allocated if the event was very rare but could not be excluded and a negligible risk score of 1 was allocated if the event was so rare and it did not merit consideration. Risk estimation was achieved by summation of the scores obtained for each exposure factor across the groups for both peri-urban and rural areas. If the summed scores for a risk practice was between 1 and 4, then the risk was considered negligible, between 5 and 8 very low risk, between 9 and 12 low risk; between 13 and 16 medium risk and between 17 and 20 high risk.

## Results

### Description of respondent’s demographics

Amongst the rural pastoralist category, a total of 38 respondents participated, and out of this number, 32 % were female. Of these respondents, 47 % of participants were between 25–30 years of age, 34 % were between 31–40 years and 19 % were above 40 years. With regard to the level of education, 45 % of the respondents had attained primary school education, 21 % had obtained secondary school level of education, and 3 % had tertiary education, while 31 % had no formal education. The peri-urban group of agro-pastoralists attracted 33 respondents, of which 46 % were female. Of these respondents from peri-urban areas, 15 % were between 25–30 years of age, 52 % between 31–40 years and 33 % were above 40 years. About 18 % of the respondents had primary school education, 49 % had secondary school level of education while 33 % had obtained tertiary level of education (see underlying data ( Onono *et al*., 2019)).

### Prevalent livestock diseases affecting herds in Kajiado County

A total of 11 livestock diseases were listed from all the eight groups of participants. There were more similarities on the livestock diseases suggested by the groups of rural pastoralists when compared to those within the peri-urban groups. However, only two of the peri-urban groups mentioned brucellosis among livestock diseases that affected their flocks and herds and none from rural pastoralists ever mentioned brucellosis. According to these pastoralists, livestock diseases which were most prevalent included contagious caprine pleuropneumonia, lumpy skin disease, and foot and mouth disease and these diseases were described to have a high impact on livestock production (Z > 1.96) ( Table 1). Amongst all listed livestock diseases, zoonoses (Anthrax and brucellosis) were ranked low, which could be due to the fact that these groups of pastoralist lack knowledge and awareness of the impact of these livestock diseases on their wellbeing and health. The level of agreement across these groups of pastoralists on rank orders for the livestock diseases based on their impact of wellbeing and health was considered weak, with the calculated Kendall’s coefficient of concordance ‘W’ estimated at 0.007. The high impact of these livestock diseases on mortality was reported for East Coast fever and contagious caprine pleuropneumonia, while milk yield was mostly affected by foot and mouth disease, lumpy skin disease, heart water, East Coast fever and diarrhea. Increased incidences of abortions were due to infection with foot and mouth disease, lumpy skin disease, contagious caprine pleuropneumonia and East Coast fever. The pastoralists from peri-urban areas associated brucellosis with an increased level of abortions in herds and flocks while anthrax was associated with high morbidity rates in livestock herds. Rank orders for livestock diseases according to pastoralists are presented as underlying data ( Onono *et al*., 2019).

**Table 1.** Ranks of livestock diseases based on their impact according to pastoralists.

Livestock diseases	Median	Mean rank	Z-score
Contagious caprine pleuropneumonia	0.40	83.50	4.53
Lumpy skin diseases	0.13	61.50	1.97
Foot and mouth disease	0.18	55.50	1.28
Diarrhea	0	45.50	0.12
Anthrax	0	39.00	-0.63
Heart Water	0	36.50	-0.93
Brucellosis	0	36.00	-0.99
East coast fever	0	36.00	-0.99
Pneumonia	0	35.00	-1.10
Eye infection	0	30.50	-1.63
Pestes des petits ruminants	0	30.50	-1.63

### Qualitative risk assessment for pastoralists’ exposure to brucellosis

Hazard identification was achieved through review of published literature under the different farming systems in Kenya (see underlying data ( Onono *et al*., 2019)). Various *Brucella species* had been detected in livestock raised in different farming systems in Kenya, and also in humans through serological tests ( Table 2). Antibodies of *B. abortus* had been detected in bovine milk through milk ring tests and Elisa test ( Kang’ethe *et al.,* 2004). According to a recent review by Njeru *et al.* (2016), *B. abortus* and *B. melitensis* had been isolated in cattle and humans and *B. suis* in rodents. Pastoralists listed various practices which may act as possible exposure factors for transmission of brucellosis from livestock to members of the community. These included handling birth products without protective gear while assisting livestock during birth process and consumption of unpasteurized milk, which were associated with high risk of exposure by household members to brucellosis among the rural pastoralists. These practices would increase exposure to Brucella pathogens and therefore increasing the risk of its transmission from livestock to household members. Within the rural community, young children were often drinking fresh raw milk and colostrum which were perceived to enhance their immunity against infections, which is also a potential risk factor for infection.

**Table 2.** Risk assessment for exposure to brucellosis in the pastoralist’s production system of Kajiado County.

Risk analysis steps	Description of evidence for risk profile	Estimates of epidemiological data	Systems affected	Authors
Hazard identification	Sero-Prevalence of Brucella in animals and man.	17% in man (n=174), 13% in goats (n=400), 11% in cattle (n= 200)	Pastoralists system in North Turkana	Nanyende, 2007 (MSc Thesis)
	Sero-prevalence of brucellosis in animals and man	Overall sero-prevalence for the three counties (Kajiado, Kiambu and Marsabit) were:16% in humans and 8% in animals; human and livestock sero-prevalence was 3 and 4 times higher in Marsabit than in Kajiado, which was 6 and 3times higher than Kiambu respectively	Extensive production system (Kajiado and Marsabit) and intensive production system (Kiambu)	Ogola *et al.* (2014).
	Prevalence of Brucella in cattle milk;	Overall prevalence of 4.9% by indirect ELISA and 3.9% by milk ring test (MRT) at consumer level and 2.4% -ELISA and 3.4% - MRT at informal market.	Milk from Extensive production system (Narok and Nakuru) and intensive production system (Nairobi and Kiambu	Kang’ethe *et al.* (2007)
Hazard characterisation	Types of Brucella organisms isolated in Kenya: *Brucella* *abortus in cattle milk* *Brucella melitensis* biovar 1 and *Brucella abortus* biovar 3 isolated in cattle	Milk sampled from urban consumers in Nairobi and Nakuru showed prevalence of 4.7% (n=10) by MRT and 5.1% (n=11) by ELISA; and from Rural consumers: 3.2% (n=7) by MRT and 4.6% (n=10) by ELISA. *Brucella melitensis* biovar one isolated from cattle milk from Central Kenya, and *Brucella abortus* biovar 3 from aborted foetal materials from cattle in Central and Eastern provinces of Kenya	Milk from Extensive production system (Narok and Nakuru) and intensive production system (Nairobi and Kiambu; Central province is characterized by intensive production system while Eastern is mainly characterised by semi- extensive production system	Kang’ethe *et al.* (2007) Muendo *et al.* (2012)
Exposure assessment	Exposure factors were: • Consumption of unpasteurized milk • Consumption of raw meat • Consumption of raw blood • Handling infected aborted materials or assisting animals during parturition without protective gloves		Peri-urban pastoralism system and rural pastoralism system	Current study
Risk characterization and estimation	Risk of human infection with Brucella organisms is higher in rural pastoralism than in peri- urban areas		Peri-urban areas and rural pastoralism system	Current study

Key: number of study units or samples which were included for specific studies which were reviewed (n); Masters of Science (MSc); milk ring test (MRT); and enzyme linked immunosorbent assay (ELISA)

Within the peri-urban group, the risk associated with exposure to brucellosis through handling of birth products without protective gear and drinking of raw fresh milk was considered to be low. These peri-urban pastoralists kept only a few livestock and they did not keep mixed livestock species. Moreover, the majority also relied on professional veterinary services during delivery or treatment of sick livestock, hence they were less exposed to brucellosis infection through handling of infected birth products. Besides, these pastoralists often used pasteurized milk, while others would always boil the raw milk from their livestock but for those households who did not own livestock, they often boiled the milk they would purchase from local shops or neighboring farms before using it within the household.

Consumption of raw meat and blood were associated with medium risk of brucellosis infection among the rural pastoralists. Consumption of raw blood was often practiced during traditional ceremonies like marriage and rites of passage when young Maasai boys are initiated to adulthood, during which time livestock were slaughtered at home. Pastoralists reported that kidneys from livestock and occasionally the liver would be consumed in their raw state during slaughtering process. This practice of home slaughter was prevalent amongst rural pastoralists due to a lack of established slaughter premises where they lived, and the long distances they would have to walk to get to the nearest marketplaces where slaughter facilities were established. The risk of exposure to brucellosis was, however, ranked low because only a few people within households were engaged in drinking the raw blood or eating the raw meat from kidneys and the liver.

Based on the risk estimation criteria, the risk of exposure to brucellosis was categorized as high amongst the rural pastoralists when compared to peri-urban pastoralists. This was due to cultural practices around consumption of fresh raw milk, raw blood and meat, and handling of birth products without protection while assisting livestock during delivery. Based on the criteria for risk estimation, consumption of fresh raw milk had a score of 19 for the rural pastoralists group and only 10 for the peri-urban group; eating of raw meat had a score of 14 for rural pastoralists and 7 for the peri-urban group; drinking of raw blood was scored 15 for the rural pastoralists and only 6 for the peri-urban group; and finally, handling of birth products without gloves while assisting livestock delivery was scored 20 for the rural pastoralists and 11 for the peri-urban group ( Table 3). Based on this criterion, risk of brucellosis infection in the rural pastoralists was high for handling birth products without protectives and drinking of fresh raw milk; while the drinking of blood and eating raw meat were rated to be of medium risk. For pastoralist within the peri-urban areas, handling of birth products and consumption of raw milk were ranked as low risk, while for consumption of raw meat and blood the risk was ranked as very low.

**Table 3.** Scores allocated per risk factor for contracting brucellosis by pastoralists in Kajiado.

*Sub-county*	*Category* *of system*	*Name of* *group*	*Drinking* *Unpasteurized milk*	*Eating* *Raw meat*	*Drinking* *Raw blood*	*Handling birth products* *without gloves*
*Kajiado central*	*Rural*	*Lorngosua*	++++	+++	+++	+++++
	*Rural*	*Ilpatimaro*	+++++	++++	++++	+++++
	*Rural*	*Meto*	+++++	++++	++++	+++++
	*Rural*	*Kumpa*	+++++	+++	++++	+++++
*Kajiado east*	*Peri-urban*	*Sholinke*	++	+	+	++
	*Peri- urban*	*Kitengela*	++	+	+	++
	*Peri –urban*	*Olturoto*	+++	+++	++	++++
	*Peri- urban*	*Kisaju*	+++	++	++	+++
*Total scores for rural households*			19	14	15	20
*Total scores for peri-urban households*			10	7	6	11

Key: +++++ = high risk score; ++++ = medium risk score; +++ = low risk score; ++ = very low risk score; + = negligible risk score

## Discussion

This study identified livestock diseases which negatively impacted on livelihoods of pastoralists within Kajiado County, through reduction in milk yield, increased mortality rates, increased morbidity rates and abortions in herds and flocks. In addition, zoonoses including brucellosis and anthrax were identified by communities as a threat both to their wellbeing and health. According to the pastoralists, livestock diseases that were significantly impacting on their livelihoods included contagious caprine pleuropneumonia, Lumpy Skin disease and foot and mouth disease, which were ranked high. These results agree with findings of a previous report which was done in Narok County, Kenya, where foot and mouth disease and lumpy skin disease were identified amongst other diseases to be most prevalent and also had negative impacts on family incomes ( Onono *et al.,* 2013). In addition to occurrence of these livestock diseases which affected levels of productivity, their knowledge on occurrence of brucellosis had previously been corroborated by findings from studies that have reported the disease in herds and flocks through serological testing and microbiological methods. According to a review of the literature on studies that were carried out in similar pastoralist systems in Kenya, brucellosis had been reported to occur either due to *B. melitensis* or *B. abortus* ( Kang’ethe *et al.,* 2007; Muendo *et al.,* 2012; Ogola *et al.,* 2014). However, the pastoralists had only ranked brucellosis 7 ^th^ out of the 11 livestock diseases that were identified to affect their livelihoods. Another zoonosis identified by the pastoralist was anthrax and it was ranked 5 ^th ^amongst all the livestock diseases affecting their wellbeing and health. These results reveal the lack of knowledge on impact of zoonoses to their livelihoods and health. Indeed the findings are not surprising based on the findings of a previous study which had reported the nature of inconsistencies in level of knowledge of zoonoses among pastoralists living in the Northern parts of Cameroon ( Moritz *et al.,* 2013). Similarly, the knowledge of the community on brucellosis in animals with regard to its clinical signs, transmission patterns and control was very poor when compared to that in humans. This finding is similar to that by Adesokan *et al.* (2013), who reported poor knowledge of brucellosis amongst a pastoralist community in Nigeria. But another study by Buhari *et al*. (2015), had reported a high level of knowledge on brucellosis in cattle when compared to infection in man, amongst a specific group of pastoralists in northern Nigeria. No explanation was provided for the cause of this apparent difference in knowledge amongst pastoralists in northern Nigeria, but it can be hypothesized that the community had obtained some form of training through extension agents on the importance and significance of brucellosis control in livestock. Indeed, the pastoralists from northern Nigeria are reportedly reliant on media teachings on improved livestock production which could explain the rise in levels of awareness on this disease. In the current study, only one group of pastoralists reported abortions as a clinical sign associated with occurrence of brucellosis in animals. This finding is consistent with reports from other studies that have documented lack of knowledge by pastoralists in northern Uganda on the clinical manifestation of brucellosis in livestock through abortions ( Kansiime *et al.,* 2014). The occurrence of brucellosis in herds and flocks is a serious public health threat to the people who are in contact with these livestock. Indeed, clinical disease in human have been reported in various parts of Kenya ( Njeru *et al.,* 2016). Furthermore, the practice by pastoralists of raising mixed livestock species per farm poses a greater risk of cross infection since the Brucella species are multi-host. It has been reported that when *Brucella melitensis* establish itself in cattle herds, it poses greater health risk since localization of infection in the udder result in shedding of large quantities of the bacteria which would contaminate the environment and therefore a greater public health hazard to humans ( Díaz Aparicio, 2013; Ducrotoy *et al.,* 2017).

With regard to exposure factors for human infection, participants identified consumption of raw unpasteurized milk, blood and raw meat from infected animals as common practices by pastoralists. These were factors which were identified to highly increase the risk of exposure to people. Indeed, according to Kansiime *et al.* (2014), consumption of raw fresh milk had been associated with increased occurrence of brucellosis in Uganda, and often the health professionals in that country would educate pastoralists to avoid consumption of raw fresh milk as a measure to control the disease. Furthermore, a study performed in Ethiopia also corroborates this finding where cases of human brucellosis was reported in up to 86% of patients who were consuming raw milk ( Regassa *et al.,* 2009). The consumption of raw milk is therefore a major exposure factor for brucellosis to humans, and based on a review that has reported that raw milk consumption is rampant within sub Saharan Africa context, the general population would be at a greater risk of infection with brucellosis ( Mfinanga *et al.,* 2003). This practice also increases the risk of transmission of other zoonoses as was argued in the publication by John *et al.* (2010). Most of the pastoralists in the rural area would feed raw fresh unpasteurized milk products and sometimes colostrum directly to their young children, which they believed would boost the immunity of these children. This is a practice which has also been reported amongst the Fulani pastoralists community of northern Nigeria, who suckle milk directly from the udder of animals ( Adesokan *et al.,* 2013). These practices are generally a major risk to pastoralist community and their children for transmission and acquiring of brucellosis.

Other factors such as consumption of raw blood, kidneys and liver during traditional ceremonies and handling birth materials without protective gear were also often practised by pastoralists. This finding is consistent with results from a study which was conducted in Ethiopia, where pastoralists would often consume raw liver ( Desta, 2015; Desta, 2016a). Indeed, the consumption of uncooked meat is reportedly a common practice amongst African communities according to previous reviews ( Corbel, 2006; John *et al.,* 2010). In addition to the exposure factors discussed above, the pastoralists would often assist livestock especially cattle during deliveries without proper protective gear which increases their risk of exposure to brucellosis and other zoonoses. Indeed, in a study which was done in Jordan, less than 6% of the study participants believed that livestock owners would use protective gear while assisting animals during deliveries ( Musallam *et al.,* 2015), while other livestock owners have also been reported to handle aborted foetal materials without protective gear ( Desta, 2016b; Lindahl *et al.,* 2015). With regard to the disposal of aborted birth materials, six groups reported that they would give the aborted foetuses to dogs and another two groups would bury them. This agrees with results obtained from a study done in Jordan where about 55% of the participants would feed the aborted material to dogs and less than 10% would bury or burn these aborted materials ( Musallam *et al.,* 2015). *Brucella abortus* has been isolated in sick dogs, which demonstrate that feeding dogs with aborted foetal parts can act as reservoirs for future occurrence of disease in both livestock and man due to the close interactions between dogs, livestock and man in the pastoralist set ups ( Díaz Aparicio, 2013).

The estimated risk of exposure to brucellosis infection was high in rural areas where it was associated with handling birth products without protective gear and drinking unpasteurized milk. The pastoralists would keep large herds of livestock of mixed species and they also practiced seasonal animal breeding, therefore parturition would occur during a specific time period. In such instances, if brucellosis was present in flocks or herds, many pastoralists can get exposed in a shorter time interval given that majority of them often engaged in assisting animal deliveries and handling birth contents without protective gear, which is a possibility as was discussed by Musallam *et al.* (2015) in a study in Jordan where only 6% of respondents reportedly were using some form of protective gear while assisting on livestock delivery. Feeding of raw fresh milk and colostrum to young children to boost their immunity is a practice which requires a lot of attention, since the consequences of this practices exposes them to increased risk of contracting brucellosis, and indeed Adesokan *et al.* (2013), in their study also described the rampant use of raw fresh milk by these pastoralist communities.

Consumption of raw meat and blood were associated with medium risk of exposure amongst pastoralist living in the rural set-up. However, they still predispose communities to increased risk of exposure because they are practices that are often practiced during traditional ceremonies where many people are involved. During these traditional ceremonies, pastoralists would often slaughter animals at home without any meat inspection services. This has been reported as a common practice among African countries, which is associated with occurrence of zoonotic diseases ( Corbel, 2006; John *et al.,* 2010).

The level of risk of exposure to brucellosis within the peri-urban areas was, however, categorized as low from practices of drinking raw fresh milk, handling birth products without protective gears, and consuming raw meat and blood. This could be due to the fact that cultural practices described within rural set up are gradually being replaced by the influence of increased information on public health for the communities who have moved closer to urban set ups. Indeed, the consumption of raw fresh milk was low amongst this group since majority would boil milk before use. In addition, most households in peri-urban areas would purchase pasteurized milk. This group of pastoralists was not consuming raw meat and blood, and therefore was at a very low or negligible risk of getting exposed to brucellosis through their consumption practices.

In conclusion, the level of risk of exposure to brucellosis for pastoralists living in rural areas was high when compared to pastoralists living within peri-urban areas. This is linked to their cultural practices and how they interact with livestock products and by-products including consumption of raw fresh milk, raw blood and meat and handling birth products without protectives. Action planning to mitigate risk from these practices through public education regarding brucellosis and its exposure factors to the vulnerable communities should be established and, this will enable them know the disease and how it is transmitted. Likewise, there is need for enhanced collaboration between the Departments of Medical and Veterinary services in zoonotic disease surveillance and its control through the established government departments like the Zoonotic Disease Unit, a body created by both the Ministry of Health and Veterinary Services to respond to occurrence of zoonoses within the country.

## Data availability

### Underlying data

Figshare: KNOWLEDGE OF PASTORALISTS ON LIVESTOCK DISEASES AND EXPOSURE ASSESSMENT TO BRUCELLOSIS WITHIN RURAL AND PERI-URBAN AREAS IN KAJIADO, KENYA. https://doi.org/10.6084/m9.figshare.9848168.v5 ( Onono *et al*., 2019)

- Raw data.xls (Excel file containing focus group responses, pastoralists rankings of livestock diseases and estimates of brucellosis exposure)

### Extended data

Figshare: KNOWLEDGE OF PASTORALISTS ON LIVESTOCK DISEASES AND EXPOSURE ASSESSMENT TO BRUCELLOSIS WITHIN RURAL AND PERI-URBAN AREAS IN KAJIADO, KENYA. https://doi.org/10.6084/m9.figshare.9848168.v5 ( Onono *et al*., 2019)

- Focus group questionnaire guide.docx (Word document containing questions used in focus groups)

Data are available under the terms of the Creative Commons Zero "No rights reserved" data waiver (CC0 1.0 Public domain dedication).
