# Supplementary material for: Knowledge of pastoralists on livestock diseases and exposure assessment to brucellosis within rural and peri-urban areas in Kajiado, Kenya
Source: F1000Res. 2019 Nov 13;8:1916. [Version 1] doi: 10.12688/f1000research.20573.1 (PMC7642991; doi:10.12688/f1000research.20573.1)
Supplement: Peer review history

# Peer review: Reviewer response for version 1

**DOI:** 10.5256/f1000research.22617.r73118

**Published:** 2020-11-04

**Authors:** Gezahegne Mamo

**Affiliations:** 1 Department of Veterinary Microbiology, Immunology and Public Health, Addis Ababa University, Bishoftu, Ethiopia

**Methods:**

- Technically, the study lacks clarity as it is quite difficult to say whether the participants have knowledge on livestock diseases or not because the clinical signs alone are not enough to define a diseases like brucellosis.
- Detail of the methods used, including the major parameters used to assess the knowledge are not described. The authors should describe briefly what criteria used to say a participating group has knowledge or not,  for that supplementary file of the questionnaire should be added to the manuscript

Is the work clearly and accurately presented and does it cite the current literature?

Yes

If applicable, is the statistical analysis and its interpretation appropriate?

Yes

Are all the source data underlying the results available to ensure full reproducibility?

No source data required

Is the study design appropriate and is the work technically sound?

Partly

Are the conclusions drawn adequately supported by the results?

Partly

Are sufficient details of methods and analysis provided to allow replication by others?

Partly

Reviewer Expertise:

Zoonotic diseases (Tuberculosis, brucellosis, Anthrax), Emerging infectious diseases,

I confirm that I have read this submission and believe that I have an appropriate level of expertise to confirm that it is of an acceptable scientific standard.

# Peer review: Reviewer response for version 1

**DOI:** 10.5256/f1000research.22617.r60300

**Published:** 2020-03-11

**Authors:** Marta Guerra

**Affiliations:** 1 Bacterial Special Pathogens Branch, Division of High-Consequence Pathogens and Pathology, National Center for Emerging and Zoonotic Infectious Diseases, Centers for Disease Control and Prevention, Atlanta, GA, USA

**Methods:**

- Selection of study subjects: Unclear how participants were selected for focus groups e.g. one per household? Were participants related, which would increase similarities?

**Data collection:**

- Unclear what, if any, information was gathered per participants or was all the data obtained through the focus groups?

**Results:**

- Qualitative risk assessment: Unclear in the text what data were from the study and which were from the articles in Table 2.

**Discussion:**

- Did not see where differences in the demographics of the focus groups (sex, age) which could influence the results were discussed.

Is the work clearly and accurately presented and does it cite the current literature?

Yes

If applicable, is the statistical analysis and its interpretation appropriate?

Not applicable

Are all the source data underlying the results available to ensure full reproducibility?

Yes

Is the study design appropriate and is the work technically sound?

Yes

Are the conclusions drawn adequately supported by the results?

Yes

Are sufficient details of methods and analysis provided to allow replication by others?

Partly

Reviewer Expertise:

Epidemiology, One Health, veterinary medicine

I confirm that I have read this submission and believe that I have an appropriate level of expertise to confirm that it is of an acceptable scientific standard.
